# Supplementary material for: Exercise modifies glutamate and other metabolic biomarkers in cerebrospinal fluid from Gulf War Illness and Myalgic encephalomyelitis / Chronic Fatigue Syndrome
Source: PLoS One. 2021 Jan 13;16(1):e0244116. doi: 10.1371/journal.pone.0244116 (PMC7806361; doi:10.1371/journal.pone.0244116)
Supplement: Attachment — Submitted filename: Response to Reviewers.docx [file pone.0244116.s008.docx]

PONE-D-20-29578

Revised Title:

Exercise modifies glutamate and other metabolic biomarkers in cerebrospinal fluid from Gulf War Illness and Myalgic Encephalomyelitis / Chronic Fatigue Syndrome

**5. Responses to Reviewers**

**Reviewer #1**: The authors compared the metabolic profiles in cerebrospinal fluid from ME/CFS and GWI and sedentary control after either rest or exercise. The study is concise and sound, but some minor revision should be corrected before further publication.

1. **Line 266-272**. It is confused that the authors compared the PC aa C42:6 levels between cfs0 and STOPP (GWI post exercise) groups, because multiple factors can contribute to the differences. Though GLM used, I think it is meaningless to compare these two groups.

*Deleted.*

2. Line 281, line 315-332. The explanation of C5-OH should be careful. The C5-OH, as the metabolite of leucine converted carnitine form is different from the long chain acylcarnitine. The elevation of acyl carnitine can also attributed to increased fatty acid oxidation after exercise (Metabolism 2002 Apr;51(4):460-4.). The elevation of C5-OH may be the result of activated leucine metabolism. Previous study has showed the positive correlation between short chain acylcarnitine levels and physical performance (DOI: 10.1096/fj.202000493R).

*Thank you for the references regarding “Long- and medium-chain fatty acid oxidation is increased in exercise-trained human skeletal muscle” and “Low intramuscular levels of short-chain acylcarnitines have been found in older females compared to males”.*

*As a result, we investigated the C5-OH findings from 3 novel perspectives and added limitations and potential pitfalls:*

Line 308 to 363:

Acylcarnitine C5-OH (C3-DC-M) was higher in postexercise ***SC*** than other groups. This suggested (i) no differences between nonexercise groups, (ii) effects of exercise (***SC***>***sc0***) and (iii) differences between postexercise ***SC*** and GWI groups. …

3. The **quality of figure 1 should be improved**

*Figure 1 has reformatted. The Pearson correlation data for each group have been placed in Supplementary Information as Excel files. The 6 panels are now shown as individual color coded Excel sheets to show correlations for each metabolite.*

*Thank you for your recommendations.*

**Reviewer #2**: The manuscript entitled “Metabolic biomarkers in cerebrospinal fluid from Gulf War Illness (GWI) and Myalgic Encephalomyelitis / Chronic Fatigue Syndrome (ME/CFS)” by Baraniuk et al submitted to PLOSOne highlighted the Dysfunction in lipid metabolism may distinguish ME/CFS from GWI. Although the manuscript addresses an important question, the manuscript is not written well, lack of coherence, less organized and difficult to follow. I recommend improving the overall presentation for publication.

*We have had an external review of the paper, and have rewritten substantial portions. These are indicated by the highlighted sections of the revised manuscript.*

*The Introduction and Methods are redundant as they describe the 6 subgroups of subjects. We have reinforced the algorithm for assigning group and disease status because it is imperative to emphasize the objective route taken to define characteristics of each subgroup and then to generate new hypotheses to explain the diverse underlying pathological mechanisms.*

*The results and discussion have been edited as per Reviewer 1. This provides additional discussion of C5-OH, other acylcarnitines and potential dysfunction of metabolomics pathways.*

*Thank you for your recommendation.*
